# The Role of Macrophage Migration Inhibitory Factor in Adipose-Derived Stem Cells Under Hypoxia

**DOI:** 10.3389/fphys.2021.638448

**Published:** 2021-07-21

**Authors:** Elena Hofmann, Josefin Soppert, Tim Ruhl, Epameinondas Gousopoulos, Simona Gerra, Gabriele Storti, Yuan Tian, Markus Brandhofer, Riccardo Schweizer, Seung-Yong Song, Nicole Lindenblatt, Norbert Pallua, Jürgen Bernhagen, Bong-Sung Kim

**Affiliations:** ^1^Department of Plastic Surgery and Hand Surgery–Burn Center, University Hospital RWTH Aachen, Aachen, Germany; ^2^Institute of Biochemistry and Molecular Cell Biology, University Hospital RWTH Aachen, Aachen, Germany; ^3^Institute for Molecular Cardiovascular Research, University Hospital RWTH Aachen, Aachen, Germany; ^4^Department of Intensive Care and Intermediate Care, University Hospital RWTH Aachen, Aachen, Germany; ^5^Department of Plastic Surgery and Hand Surgery, University Hospital of Zürich, Zurich, Switzerland; ^6^Chair of Vascular Biology, Institute for Stroke and Dementia Research (ISD), LMU University Hospital, Ludwig Maximilian University of Munich (LMU), Munich, Germany; ^7^Plastic and Reconstructive Surgery, Department of Surgical Sciences, University of Rome “Tor Vergata”, Rome, Italy; ^8^Department of Plastic and Reconstructive Surgery, Yonsei University College of Medicine, Seoul, South Korea; ^9^Aesthetic Elite International–Private Clinic, Dusseldorf, Germany; ^10^Munich Cluster for Systems Neurology (SyNergy), Munich, Germany

**Keywords:** adipose-derived stem cells, macrophage migration inhibitory factor, hypoxia, cytokine, chronic wounds

## Abstract

**Background:** Adipose-derived stem cells (ASCs) are multipotent mesenchymal stem cells characterized by their strong regenerative potential and low oxygen consumption. Macrophage migration inhibitory factor (MIF) is a multifunctional chemokine-like cytokine that is involved in tissue hypoxia. MIF is not only a major immunomodulator but also is highly expressed in adipose tissue such as subcutaneous adipose tissue of chronic non-healing wounds. In the present study, we investigated the effect of hypoxia on MIF in ASCs isolated from healthy versus inflamed adipose tissue.

**Methods:** Human ASCs were harvested from 17 patients (11 healthy adipose tissue samples, six specimens from chronic non-healing wounds). ASCs were treated in a hypoxia chamber at <1% oxygen. ASC viability, MIF secretion as well as expression levels of MIF, its receptor CD74, hypoxia-inducible transcription factor-1α (HIF-1α) and activation of the AKT and ERK signaling pathways were analyzed. The effect of recombinant MIF on the viability of ASCs was determined. Finally, the effect of MIF on the viability and production capacity of ASCs to produce the inflammatory cytokines tumor necrosis factor (TNF), interleukin (IL)-6, and IL-1β was determined upon treatment with recombinant MIF and/or a blocking MIF antibody.

**Results:** Hypoxic treatment inhibited proliferation of ASCs derived from healthy or chronic non-healing wounds. ASCs from healthy adipose tissue samples were characterized by a low degree of MIF secretion during hypoxic challenge. In contrast, in ASCs from adipose tissue samples of chronic non-healing wounds, secretion and expression of MIF and CD74 expression were significantly elevated under hypoxia. This was accompanied by enhanced ERK signaling, while AKT signaling was not altered. Recombinant MIF did stimulate HIF-1α expression under hypoxia as well as AKT and ERK phosphorylation, while no effect on ASC viability was observed. Recombinant MIF significantly reduced the secretion of IL-1β under hypoxia and normoxia, and neutralizing MIF-antibodies diminished TNF-α and IL-1β release in hypoxic ASCs.

**Conclusions:** Collectively, MIF did not affect the viability of ASCs from neither healthy donor site nor chronic wounds. Our results, however, suggest that MIF has an impact on the wound environment by modulating inflammatory factors such as IL-1β.

## Introduction

Besides their substantial burden to patients and treating specialists, critical wounds also challenge global healthcare systems financially. Critical wounds are classically treated by wound dressing regimens and surgical debridements, but these measures are only partially successful. Therefore, alternative solutions are urgently needed. Regenerative medicine offers innovative approaches in this regard as it capitalizes on body-own resources to restore tissue damage. Promising developments in the field are based on the use of adipose tissue due to its easy harvest, high yields of mesenchymal stromal cells (MSCs) and its hitherto largely underestimated reparative capacity. In fact, potential regenerative medicine strategies are directed toward the regulatory function of adipose tissue and its inherent cells in body physiology. In regenerative medicine, adipose-derived stromal cells (ASCs) are of particular interest and have been studied extensively in the last two decades. ASCs are adult MSCs with rich proliferation and differentiation potential and naturally low oxygen consumption (Zuk et al., 2001). They are regarded beneficial for a multitude of regenerative therapeutic approaches such as ASC transplantation or ASC-enriched fat grafting into chronic wounds. One major characteristic of chronic wounds is a state of tissue hypoxia due to reduced tissue perfusion (Pierpont et al., 2014). Inadequate oxygenation of tissue, a phenomenon also seen in physiological conditions including strenuous exercise and high altitudes or pathologies such as obesity, cancer and ischemic diseases, stresses cells and in the long run impairs cellular function (Ruthenborg et al., 2014). However, the impact of hypoxia on MSC and ASC viability and function is still controversial. Wang et al. (2005) found no difference in the proliferation of primary human ASCs exposed to 5 or 20% oxygen tension. Fehrer et al. (2007) and Estrada et al. (2012), however, provided strong evidence that lower oxygen tension increases growth and the lifespan of ASCs and bone marrow-derived MSCs (BMSCs), respectively. These studies were seconded by Choi et al. (2014) who observed increase proliferation of primary ASCs and a specific enhancement in chondrogenic differentiation. On the contrary, Holzwarth et al. (2010) reported a decrease in the proliferation and osteogenic differentiation of BMSCs under hypoxia.

Besides the proliferating and migratory capacity of ASCs, the release of soluble factors from ASCs has been recognized as the key mechanism to exert their regenerative potential (Salgado et al., 2010). Hypoxic tension in ASCs has been shown to even enhance their therapeutic function by positively regulating their secretome (Chung et al., 2009; Choi et al., 2017).

A factor that may play a distinct role in ASC function under hypoxic conditions is the macrophage migration inhibitory factor (MIF). MIF was one of the first cytokines to be discovered over half a century ago. It was initially described as a T lymphocyte-derived factor in the context of delayed-type hypersensitivity reactions (David, 1966; Calandra and Roger, 2003). Today, MIF is known as a multifunctional chemokine-like inflammatory cytokine that plays a key role in various acute and chronic inflammatory diseases, autoimmunity, and cardiovascular conditions including atherosclerosis (Bernhagen et al., 1993; Calandra and Roger, 2003; Morand et al., 2006; Zernecke et al., 2008; Müller et al., 2012; Tilstam et al., 2017; Kang and Bucala, 2019). The physiological and pathogenic functions of MIF as well as those of the MIF homolog D-dopachrome tautomerase (D-DT/MIF-2) are mediated in a context- and cell-dependent manner via interactions with one or a combination of its receptors, i.e., CD74, CXCR2, CXCR4, or CXCR7 (Leng et al., 2003; Bernhagen et al., 2007; Zernecke et al., 2008; Tilstam et al., 2017; Kapurniotu et al., 2019). MIF is not only expressed in immune cells but also in certain parenchymal cells and various tissues (Calandra and Roger, 2003).

Notably, MIF’s high expression in adipose tissue was documented early on (Skurk et al., 2005; Kim et al., 2015a,b). MIF is considered to be an atypical chemokine and cytokine that differs from classical cytokines by several characteristics. For example, in contrast to other cytokines, MIF does not contain an N-terminal signal peptide and is rapidly released from preformed intracellular pools upon activation via several stimulants (Weber et al., 2008).

Of note, MIF has been implicated in tissue hypoxia and its expression and secretion has been shown to be induced by hypoxia in various cell types including endothelial cells and cancer cells (Oda et al., 2008; Simons et al., 2011). Moreover, we recently discovered an adipokine-like property of MIF. MIF is up-regulated in human subcutaneous adipose tissue samples harvested from the vicinity of chronic non-healing wounds when compared to adipose tissue samples from healthy donor sites (Kim et al., 2015b). Importantly, MIF secreted by inflamed adipose tissue samples impaired fibroblast proliferation in an *in vitro* wound healing assay (Kim et al., 2015b).

In the present study, we investigated the effect of hypoxia on human ASCs from healthy donor sites and the vicinity of chronic non-healing wounds. We analyzed the expression of MIF, its receptor CD74 as well as AKT and ERK phosphorylation, two common MIF signaling pathways mediating survival and apoptosis, in response to hypoxic challenge (Lue et al., 2006, 2007). Furthermore, we added recombinant MIF to normoxic and hypoxic ASCs and assessed their proliferation as well as cytokine production capacity to further evaluate a potential therapeutic value of MIF. Due to MIF’s role as an upstream regulator of multiple cytokines (Calandra and Roger, 2003), we also investigated the secretion profile of interleukin (IL)-6, IL-1β, and tumor necrosis factor (TNF)-α that have been shown to affect wound repair (Kondo and Ohshima, 1996). We hypothesized that MIF secretion is increased under hypoxia as found within the majority of chronic non-healing wounds. We also hypothesized that ASCs isolated from adipose tissue from healthy and chronic non-healing wound donor sites may react differently upon hypoxia, may have a significant impact on the release of MIF downstream cytokines, and that external MIF may stimulate ASC viability.

## Materials and Methods

### Isolation, Characterization and Cultivation of ASCs

Adipose-derived stem cells (ASCs) were isolated from human adipose tissue obtained from elective surgeries of the Department of Plastic and Reconstructive Surgery, Hand Surgery–Burn Center of the RWTH University Hospital Aachen according to a modified protocol by Zuk et al. (2001) and Pallua et al. (2009). In short, subcutaneous adipose tissue that was *en bloc* excised during surgery was immediately transferred to the laboratory. The adipose tissue samples were washed with phosphate buffered saline (PBS). Blood vessels, connective tissue and dead tissue were removed. Next, the adipose tissue samples was minced and incubated with 0.2% collagenase (type I; Worthington Biochemical, Lakewood, NJ, United States) under constant shaking for 37°D for 45 min. The collagenase digestion was stopped by Dulbecco’s modified eagle medium (DMEM)/F12 (Invitrogen, Karlsruhe, Germany) containing 10% fetal calf serum (FCS) (Gibco, Eggenstein, Germany). The digested fat was filtered through a 100 μm mesh, centrifuged at 132 × *g* for 5 min. The resulting pellet represents the stromal vascular fraction (SVF). The SVF was resuspended in a medium containing DMEM/F12 supplemented with 10% FCS, 1 ng/ml basic fibroblast growth factor (bFGF) (R&D Systems, Minneapolis, MN, United States) and 1% penicillin/streptomycin (Gibco, Eggenstein, Germany) in a humidified incubator at 37°C and 5% CO_2_ to differentiate to ASCs.

Characterization of ASCs was done according to the recognized criteria of the International Society for Cellular Therapy (ISCT) and for Adipose Therapeutics and Science (IFATS) which include plastic adherence in culture, trilinear differentiation (adipogenic, osteogenic, and chondrogenic) and marker expression (Dominici et al., 2006; Bourin et al., 2013). Upon incubation with respective differentiation media, adipogenic differentiation was confirmed by oil red O staining (Sigma-Aldrich Corporation, St Louis, MO, United States), chondrogenic differentiation by Alcian-PAS blue (Merck Millipore, Burlington, VT, United States) and osteogenic differentiation by Alizarin red staining (Sigma-Aldrich Corporation, St Louis, MO, United States) according to well-established protocols (Yoshinoya et al., 2020). By flow cytometry, following surface markers were determined on a LSR II cytometer (BD Bioscience, San Jose, CA, United States) according to earlier protocols (Pallua et al., 2018): CD31-eFluor450, CD34-FITC, CD45-PerCP-Cy5.5, CD73-PE-Cy7, CD90-PE, and CD105-APC. ASCs were negative for the hematopoietic marker CD45- and the endothelial marker CD31- but positive for the MSC markers CD34, CD73, CD90, and CD105 (Bourin et al., 2013; Yoshinoya et al., 2020).

Adipose-derived stem cells from 17 fat tissue donors (see Tables 1, 2 for closer characteristics) were included in the hypoxic versus normoxic experiment and categorized into two groups. Fat tissue donors in group A (11 donors, five male, six female, 46.82 ± 14.57 years, mean BMI 28.31 ± 3.93 kg/m^2^ underwent elective procedures. Adipose tissue samples in group B (six donors, three male, three female, mean age 47.83 ± 11.91 years, mean BMI 30.45 ± 4.43 kg/m^2^) were obtained from chronic non-healing wounds which underwent surgical wound debridement. Only viable adipose tissue from non-healing wounds was used whereas necrotic and infected parts were used for clinical sampling or discarded. Informed consent was obtained from all patients, and ethical approval was provided by the ethics committee of RWTH Aachen University (EK 213/17) and the cantonal ethics committee Zurich (BASEC-Nr 2019-00389). Experiments were conducted in accordance with the Declaration of Helsinki Principles.

**TABLE 1:** Details of healthy adipose tissue samples in group A.

Number	Age	Sex	BMI	Location	Applied Assays
1	38	Male	29.07	Lower leg	MIF ELISA
2	56	Male	26.12	Abdomen	Proliferation, MIF ELISA
3	31	Male	24.38	Breast	Proliferation, MIF ELISA
4	37	Male	25.25	Abdomen	Proliferation, MIF ELISA, MIF Dose: Proliferation and Western Blot
5	65	Female	36.33	Abdomen	MIF Dose: Proliferation and Western Blot
6	36	Female	29.3	Upper thigh	MIF stimulation, ELISA (IL-6, IL-1β, TNF-α), Western Blot (CD74, HIF-1α)
7	76	Male	28.9	Abdomen	MIF stimulation, ELISA (IL-6, IL-1β, TNF-α), Western Blot (CD74, HIF-1α)
8	45	Female	22.2	Abdomen	MIF stimulation, ELISA (IL-6, IL-1β, TNF-α), Western Blot (CD74, HIF-1α)
9	30	Female	32.7	Abdomen	MIF stimulation, ELISA (IL-6, IL-1β, TNF-α), Western Blot (CD74, HIF-1α)
10	51	Female	27.5	Abdomen	MIF stimulation, ELISA (IL-6, IL-1β, TNF-α),
11	50	Female	29.7	Upper thigh	MIF stimulation, ELISA (IL-6, IL-1β, TNF-α),

**TABLE 2:** Details of adipose tissue samples from chronic non-healing wounds in group B.

Number	Age	Sex	BMI	Location	Applied Assays
12	27	Male	25.9	Upper leg	Proliferation, MIF ELISA, Western Blot
13	51	Female	27.82	Abdomen	Proliferation, MIF ELISA, Western Blot
14	56	Female	35.75	Upper leg	Proliferation, MIF ELISA, Western Blot, MIF Dose: Proliferation and Western Blot
15	41	Female	29.5	Abdomen	MIF neutralization, ELISA (IL-6, IL-1β, TNF-α), Western Blot (CD74, HIF-1α)
16	59	Male	27.5	Upper leg	MIF neutralization, ELISA (IL-6, IL-1β, TNF-α), Western Blot (CD74, HIF-1α)
17	53	Male	36.2	Upper leg	MIF neutralization, ELISA (IL-6, IL-1β, TNF-α), Western Blot (CD74, HIF-1α)

### Hypoxic Treatment of ASCs

A commercially available hypoxia chamber (Coy Laboratory Products, Grass Lake, MI, United States) was continuously flooded with 95% nitrogen (N_2_) and 5% CO_2_ to maintain a hypoxic environment below 1% O_2_. Deoxygenation of growth and starvation media supplemented with 10 and 1% FCS, respectively, was achieved as previously described (Simons et al., 2011). ASCs at passage three were seeded at a density of 10,000 cells/cm^2^ in growth medium. All cells were incubated at 37°C and 5% CO_2_ overnight to facilitate cell adherence before hypoxic or normoxic treatment.

### Detection of Cell Viability Under Normoxia and Hypoxia

The viability of ASCs was documented by microscopic photographs and quantified using Trypan Blue staining (Invitrogen, Karlsruhe, Germany) and a TC20 Automated Cell Counter (Bio-Rad, Munich, Germany) according to the manufacturer’s guidelines. The metabolic activity of ASCs was quantified using PrestoBlue (Invitrogen, Karlsruhe, Germany) according to the manufacturer’s manual. Fluorescence was measured at 560 nm excitation and 590 nm emission with wavelength correction by a Wallac Victor 1420 multilabel counter.

### Quantification of Cytokine Secretion by Enzyme-Linked Immunosorbent Assay (ELISA)

Levels of MIF, IL-6, IL-1β, and TNF-α in the supernatants of ASCs were measured by enzyme-linked immunosorbent assay (ELISA) (human MIF, human IL-6, human IL-1 beta/IL-1F2 and human TNF-alpha Duo Set ELISA kit, R&D Systems, Minneapolis, MN, United States) according to the manufacturer’s manual. Color intensity was measured using a Wallac Victor 1420 multilabel counter or EnSpire Multimode Plate Reader (PerkinElmer, Rodgau, Germany) set at 450 nm with wavelength correction.

### Analysis of the Expression of MIF, CD74, HIF-1α, and AKT and ERK Signaling Using Western Blot

The expression levels of hypoxia-inducible transcription factor-1α (HIF-1α), intracellular MIF, its receptor CD74, as well as the phosphorylation levels of AKT and ERK, and finally, tubulin for total protein standardization was performed by Western blot as previously described (Lue et al., 2006).

Cell lysates of ASCs at indicated measurement points (see section “Results”) were acquired using 1× LDS/DTT buffer [25% (v/v) NuPAGE lithium dodecyl sulfate (LDS) sample buffer (Invitrogen, Karlsruhe, Germany), 50 mM dithiothreitol (DTT) (Sigma-Aldrich, Munich, Germany), bidistilled water (ddH_2_O)]. Sodium dodecyl sulfate-polyacrylamide gel electrophoresis (SDS-PAGE) for MIF (12.5 kDa) and CD74 (40 kDa) or HIF-1α (120 kDa), AKT (60 kDa), and ERK-1/2 (42–44 kDa) analysis using 15 or 10% polyacrylamide gels, respectively, and Western blotting was performed according to standard protocols (Lue et al., 2006). The following primary antibodies were used: rabbit anti-HIF-1α (D1S7W; Cell Signaling Technology, Beverly, MA, United States), goat anti-CD74 (C-16 or LN-2) (Santa Cruz Biotechnology, Heidelberg, Germany), mouse anti-αTubulin (B512; Sigma-Aldrich, Munich, Germany or B-7, Santa Cruz Biotechnology, Heidelberg, Germany), mouse anti-ERK1/2 (C-9) and anti-pERK-1/2 (12D4; Santa Cruz Biotechnology, Heidelberg, Germany), rabbit anti-AKT (11E7) and anti-pAKT (Ser473; Cell Signaling Technology, Beverly, MA, United States). The polyclonal rabbit anti-MIF antibody (Ka565) was previously described (Schober et al., 2004). Mouse anti-rabbit Peroxidase IgG (Jackson ImmunoResearch, Ely, United Kingdom), rabbit anti-goat-HRP (Pierce Biotechnology, Rockford, IL, United States), donkey anti-mouse-HRP (Abcam, Cambridge, MA, United States), goat anti-mouse-HRP (Abcam, Cambridge, MA, United States) and donkey anti-rabbit-HRP (GE Healthcare, Munich, Germany) were employed as secondary antibodies. Blotted bands were detected using the LAS-3000 image reader (Fujifilm, Düsseldorf, Germany) and quantified by AIDA image analyzer software (Raytest Isotopen GmbH, Berlin, Germany) or Image J (1.53a, National Institutes of Health, United States).

### MIF Stimulation and Neutralization Experiment

Briefly, 10,000 ASCs/cm^2^ were incubated in growth medium overnight at 37°C and 5% CO_2_. To study the effect of recombinant MIF on the viability of ASCs, cells were subjected to hypoxic versus normoxic treatment in the presence of different MIF concentrations (100, 250, and 500 ng/ml, as determined in a prior dose response scouting experiment) that were added at 0, 24, 48, and 72 h. The influence of exogenous MIF on cytokine release and expression of HIF-1α and intracellular CD74 were studied by using ASCs stimulated with 100 ng/ml recombinant MIF at normoxic or hypoxic conditions for 24 h. Biologically active, recombinant human MIF was prepared as described previously (Bernhagen et al., 1994; Kleemann et al., 2000). For neutralization experiments, 8000 nM of anti-MIF monoclonal antibody NIH/IIID.9 was applied at normoxic or hypoxic conditions for 24 h. The control group received PBS in all experiments.

### Statistical Analysis

Statistical analysis was performed using GraphPad Prism version 8 (La Jolla, CA, United States). Samples were tested for normality using Shapiro–Wilk test. For experiments comparing the effect of normoxic and hypoxic condition either over time or following stimulation with vehicle or recombinant MIF on the functional biology of ASCs, two-way ANOVA was applied and corrected for multiple comparisons using the Holm-Šídák method. The effect of the MIF-dose response on the activation of stress kinases was statistically evaluated using one-way ANOVA corrected by Holm-Šídák’s multiple comparisons test. Unpaired, two-sided *t*-tests were performed for MIF neutralization experiments in hypoxic ASCs isolated from chronic non-healing wounds. Results are presented as mean values with the standard error of the mean (SEM). Asterisks indicate statistical significance, considered as a *p*-value below 0.05.

## Results

### ASCs Meet ISCT/IFATS Criteria

Adipose-derived stem cells were characterized according to ISCT and IFATS criteria on MSCs. ASCs in cultured showed plastic adherence and differentiated into the osteogenic, chondrogenic and adipogenic line (Supplementary Figure 1). Furthermore, ASCs were negative for the markers CD31/CD45 but positive for the stem cell markers CD34, CD73, CD90, and CD105 (Supplementary Figure 2).

### Hypoxia Reduces the Proliferation of ASCs

**FIGURE 1:**
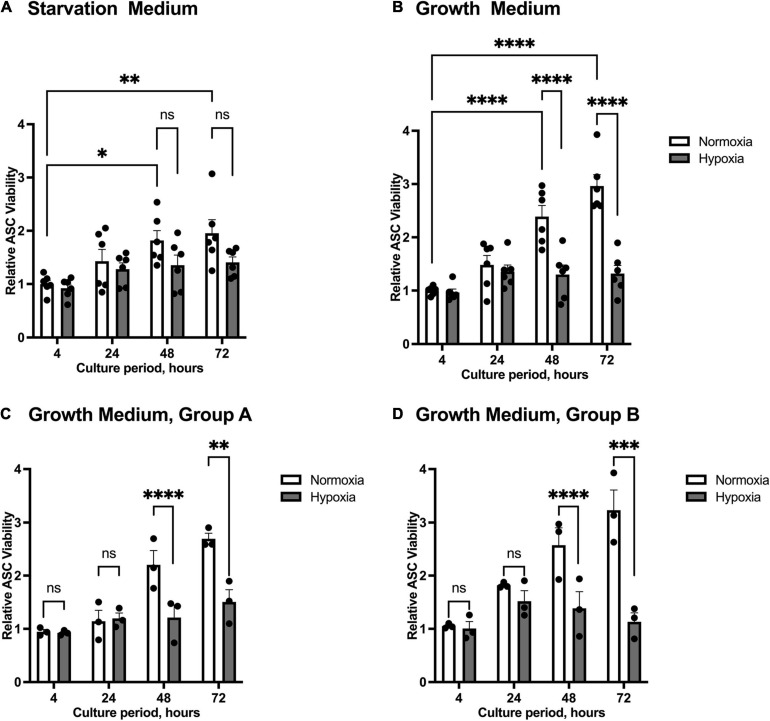
Viability of ASCs under normoxic and hypoxic conditions. Cells were cultured in starvation or growth medium at normoxic oxygen levels of 21% O_2_ or hypoxic oxygen levels of <1% O_2_. Viability of ASCs was measured by Trypan Blue staining and counting in relation to the baseline value at 4 h normoxia, set to one. Graphs display pooled effects of ASCs isolated from patient Group A and B cultured in **(A)** starvation medium (*n* = 6) and **(B)** growth medium (*n* = 6). ASCs harvested from **(C)** healthy donor sites (Group A, *n* = 3) and **(D)** chronic, non-healing wounds (Group B, *n* = 3) exert similar proliferation behavior when cultured in growth medium. Data are represented as mean values ± SEM (two-way ANOVA, Holm-Šídák’s multiple comparisons test, ^∗^*p* < 0.05, ^∗∗^*p* < 0.01, ^∗∗∗^*p* < 0.001, ^****^p < 0.0001).

We firstly tested the effect of hypoxia on the viability of ASCs. We examined the effect of hypoxia and different cell media composition on cell survival using starvation medium to mimic the state of nutrient deprivation in wounds, and growth medium to exclude effects of nutrient deficiency. Cells from group A (healthy donor sites) and group B (chronic non-healing wounds) were both examined. ASC proliferation was inhibited at oxygen levels below 1%, while the number of ASCs increased significantly at 48 h and 72 h under normoxic conditions. Compared to growth medium, nutrient deprivation seems to slow down the proliferation rate of normoxic ASCs (Figures 1A,B). Thus, proliferation of ASCs maintained in growth medium and under normoxia were significantly increased at 48 and 72 h compared to those kept under hypoxic conditions. The hypoxia-associated restriction in cell proliferation was independent of patient group (Figures 1C,D). Together, the experiment showed that hypoxic treatment reduces cell proliferation.

### Hypoxic Treatment Induces MIF Secretion

**FIGURE 2:**
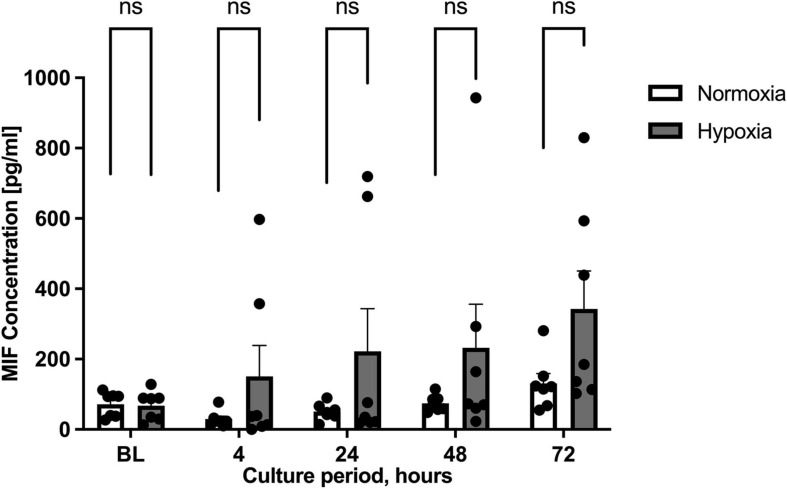
Migration inhibitory factor (MIF) secretion levels under normoxic and hypoxic conditions. Cells were cultured in starvation medium at normoxic oxygen levels of 21% O_2_ or hypoxic oxygen levels of <1% O_2_. Supernatants of ASCs were collected at indicated measuring points and ELISA was performed to determine MIF secretion by ASCs under normoxic and hypoxic conditions. Baseline levels (BL) were obtained at the starting point 0 h. The graph displays pooled results from patients group A (healthy donor sites) and group B (chronic non-healing wounds). Analyses were performed for seven biological replicates (*n* = 7). Data are mean values ± SEM (two-way ANOVA, Holm-Šídák’s multiple comparisons test, not significant).

We next wished to study whether the effect of hypoxia altered MIF secretion. MIF secretion levels in the supernatants of ASCs cultured under hypoxic conditions were compared to those under normoxic conditions. As we did not differentiate between patient group A and B, results of both specimens were pooled in this part. Starvation medium was utilized to limit the possible interference of FCS in the ELISA (Kragstrup et al., 2013). Figure 2 illustrates that MIF secretion was increased by the hypoxic challenge at all time intervals studied. Maximum secretion was observed 72 h after the start of hypoxia with an accumulated MIF concentration of 300 pg/ml, corresponding to a two-fold increase over MIF secretion under normoxic conditions (not significant). The results indicate that MIF secretion may be elevated by hypoxic challenge of ASCs.

### Hypoxia-Induced MIF Secretion Is Elevated in ASCs From Adipose Tissue of Chronic Non-healing Wounds

**FIGURE 3:**
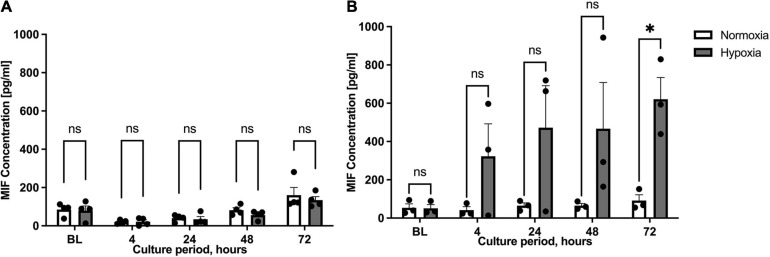
Migration inhibitory factor (MIF) secretion levels under normoxic and hypoxic conditions in patient groups A and B. Cells were cultured in starvation medium at normoxic oxygen levels of 21% O_2_ or hypoxic oxygen levels of 1% <O_2_. Separate replicates were cultured for indicated culture periods for independent measurements. Supernatants of ASCs were collected at indicated measuring points and ELISA was performed to determine MIF secretion by ASCs under normoxic and hypoxic conditions. Baseline levels (BL) were obtained at the starting point 0 h. Fat donors were arranged in two groups and analyses were performed for four or three biological replicates, respectively. Group A included healthy adipose tissue samples and group B adipose tissue samples from chronic non-healing wounds. Results are displayed for **(A)** patient group A (*n* = 4) and **(B)** patient group B (*n* = 3). Data are mean values ± SEM (two-way ANOVA, Holm-Šídák’s multiple comparisons test, ^∗^*p* < 0.05).

Previous studies described the up-regulation of MIF expression in human subcutaneous adipose tissue specimens from chronic non-healing wounds when compared to healthy donor sites (Kim et al., 2015b). We therefore compared hypoxia-stimulated MIF secretion between ASCs isolated from healthy (group A) and inflamed adipose tissue specimens (group B) next. ASCs from healthy adipose tissue samples (group A) were characterized by low MIF secretion levels under hypoxic conditions with no statistical difference to the normoxic control (Figure 3A). By contrast, hypoxia-induced MIF secretion in ASCs isolated from group B individuals with chronic non-healing wounds was markedly increased when compared to normoxic treatment reaching statistical significance after 72 h (*p* < 0.05) (Figure 3B). The results indicate that hypoxia-induced MIF secretion is specifically increased in ASCs samples from adipose tissue samples of chronic non-healing critical wounds whereas hypoxia has no influence on the MIF secretion from ASCs derived from healthy donor sites.

### MIF Secretion and Expression Under Hypoxia Correlates With CD74 Expression Levels in ASCs Isolated From Adipose Tissue of Chronic Non-healing Wounds

**FIGURE 4:**
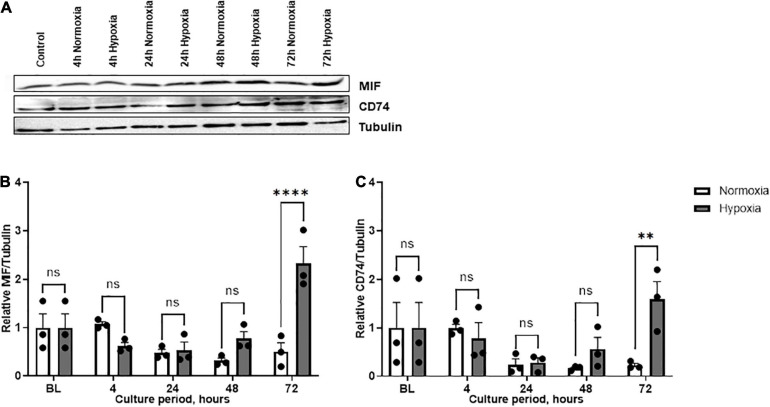
Expression levels of MIF and CD74 under normoxia and hypoxia in patient group B. Group B included three adipose tissue specimens from chronic non-healing wounds, which exhibited high MIF secretion in the hypoxic culture of ASCs. Cells were cultured in growth medium at normoxic oxygen levels of 21% O_2_ or hypoxic oxygen levels of <1% O_2_. Cell lysates were obtained at indicated measuring points. Western blot analysis was performed and evaluated using the AIDA software. Tubulin was detected for total protein standardization. **(A)** Blots are representative of all Western blots performed to detect levels of MIF, CD74, and tubulin. Analyses were performed for three biological replicates, respectively (*n* = 3). The graphs display **(B)** relative MIF/tubulin and **(C)** relative CD74/tubulin normalized to the baseline value (BL) at 0 h, set to one. Data are mean values ± SEM (two-way ANOVA, Holm-Šídák’s multiple comparisons test, ^∗∗^*p* < 0.01, ^****^p < 0.0001).

Based on the finding that MIF secretion was specifically and strongly elevated in ASCs isolated from chronic non-healing adipose tissue, ASCs from patient group B were further scrutinized. Western blot analysis was used to confirm the effect of hypoxia on MIF secretion and to examine expression of CD74. Enhanced MIF secretion was accompanied by significantly elevated cellular MIF expression levels under hypoxic culture of ASCs from chronic non-healing wounds when compared to normoxic conditions (Figure 4). This difference was statistically significant at 72 h after starting the hypoxic challenge (*p* < 0.0001). The hypoxia-triggered up-regulation of MIF at later time points was mirrored by the CD74 expression profile, reaching statistical significance at 72 h hypoxia (*p* < 0.01). The results demonstrate a potential correlation between hypoxia-induced MIF secretion and CD74 expression in ASCs isolated from chronic non-healing adipose tissue of patient group B.

### Elevated MIF Secretion After Hypoxic Challenge Is Accompanied by Altered ERK Signaling in ASCs Isolated From Adipose Tissue of Chronic Non-healing Wounds

**FIGURE 5:**
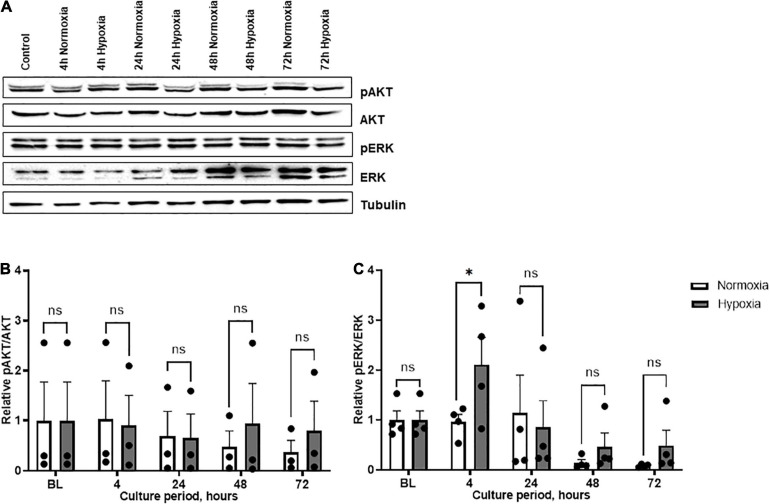
AKT and ERK phosphorylation in ASCs under normoxia and hypoxia in patient group B. Group B included adipose tissue samples from chronic non-healing wounds, which exhibited high MIF secretion in the hypoxic culture of ASCs. Cells were cultured in growth medium at normoxic oxygen levels of 21% O_2_ or hypoxic oxygen levels of <1% O_2_. Cell lysates were obtained at indicated measuring points and Western blot analysis was performed and evaluated using the AIDA software. Tubulin was detected for total protein standardization. **(A)** Blots are representative of all Western blots performed to detect levels of phosphorylated AKT (pAKT), AKT, phosphorylated ERK (pERK), and ERK. Analyses were performed for three biological replicates (*n* = 3). The graphs display **(B)** relative pAKT/AKT and **(C)** relative pERK/ERK were normalized to the baseline value at 0 h, set to one. Data are mean values ± SEM (two-way ANOVA, Holm-Šídák’s multiple comparisons test, ^∗^*p* < 0.05).

The relationship between hypoxia-induced MIF secretion and cell signaling was further examined in ASCs from patient group B. We chose to analyze the PI3K/AKT and MAPK/ERK signaling pathways as they are prominently involved in cell survival and have been associated with MIF and its receptors (Zernecke et al., 2008). AKT phosphorylation did not change significantly under hypoxia, neither over the time course nor when compared to normoxic conditions, although a trend toward increased hypoxia-induced AKT phosphorylation was seen at 72 h (Figures 5A,B). ERK activation gradually attenuated over the time course, both under hypoxic and normoxic conditions. Overall, pERK/ERK ratios were increased at 4, 48, and 72 h of hypoxic challenge under hypoxia as compared to normoxia with a significant increase at 4 h hypoxia (*p* < 0.05) (Figures 5A,C). The results suggest a link between the hypoxia-induced increase in MIF secretion and the altered CD74/ERK signaling response in ASCs.

### Treatment With Exogenous Recombinant MIF May Dose-Dependently Activate AKT and ERK Signaling in ASCs, but Does Not Alter ASC Viability Under Hypoxia

**FIGURE 6:**
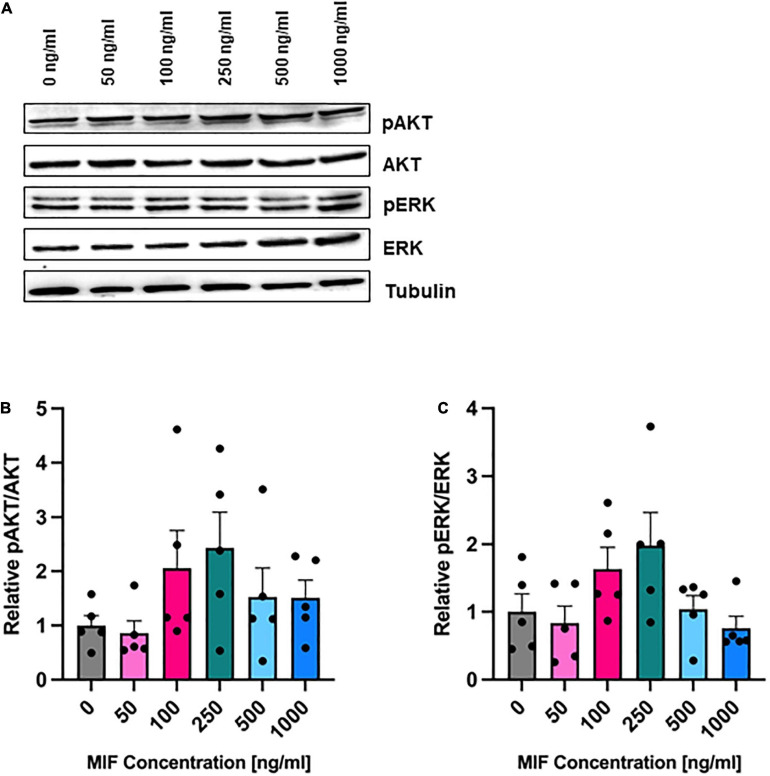
AKT and ERK phosphorylation after MIF stimulation. ASCs were cultured in growth medium at normoxic oxygen levels of 21% O_2_ for 24 h and then stimulated with various concentrations of exogenous MIF for 15 min. The control (0 ng/ml) did not receive any exogenous MIF. Cell lysates were obtained and Western blot analysis was performed and evaluated using the AIDA software. Tubulin was detected for total protein standardization. **(A)** Blots are representative of all Western blots performed to detect levels of AKT and ERK activation. Analyses were performed on five biological replicates (*n* = 5). Graphs display **(B)** relative pAKT/AKT and **(C)** relative pERK/ERK normalized to the activation level of unstimulated cells set to one. Data are mean values ± SEM (one-way ANOVA, Holm-Šídák’s multiple comparisons test, not significant).

To directly test the effects of MIF stimulation, we incubated ASCs with recombinant MIF. MIF dose-dependently enhanced the levels of phosphorylated ERK and AKT, with a maximal effect of two-fold up-regulation seen at 250 ng/ml MIF, but the MIF dose effect did not reach statistical significance (not significant; Figure 6). This observed trend is in line with previous reports in other cell types (Lue et al., 2007; Zhang et al., 2012).

**FIGURE 7:**
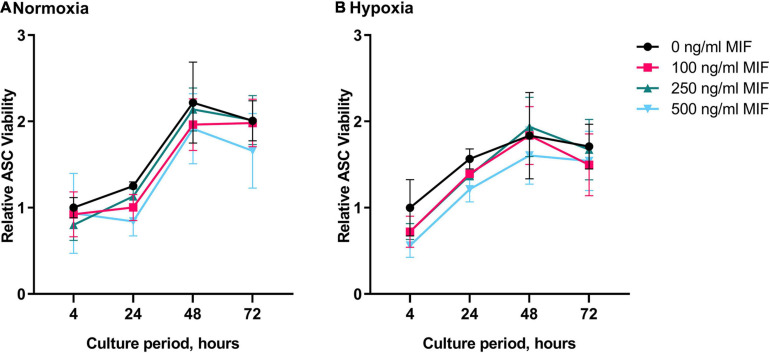
Viability of ASCs under normoxic and hypoxic conditions after MIF stimulation. Cells were stimulated with 100, 250, or 500 ng/ml of recombinant MIF and cultured in growth medium at normoxic oxygen levels of 21% O_2_ or hypoxic oxygen levels of <1% O_2_. Treatment with exogenously added MIF was repeated every 24 h of culture. Viability of ASCs measured by Presto Blue assay was calculated in relation to the baseline value (BL) of the control (0 ng/ml) that did not receive any exogenous MIF at 4 h normoxia, set to one. Analyses were performed on three biological replicates (*n* = 3). Results are displayed for ASCs cultured in **(A)** normoxia and **(B)** hypoxia. Data are mean values ± SEM (two-way ANOVA, Holm-Šídák’s multiple comparisons test, not significant).

We next recorded the viability of ASCs under hypoxic and normoxic conditions and tested the effect of recombinant MIF. Since AKT and ERK are well-known mediators of proliferation, we next tested the effect of recombinant MIF on the viability of ASCs under hypoxic and normoxic conditions. While the viability of ASCs increased in a time-dependent manner with a maximum observed after 48 h, no effect of hypoxia or MIF treatment was noted (Figure 7). In summary, recombinant MIF did not have any effects on cell viability, although it appears to lead to AKT and ERK phosphorylation in a dose-dependent manner.

### Recombinant MIF and Hypoxia Synergistically Enhance HIF-1α Protein Expression in ASCs

**FIGURE 8:**
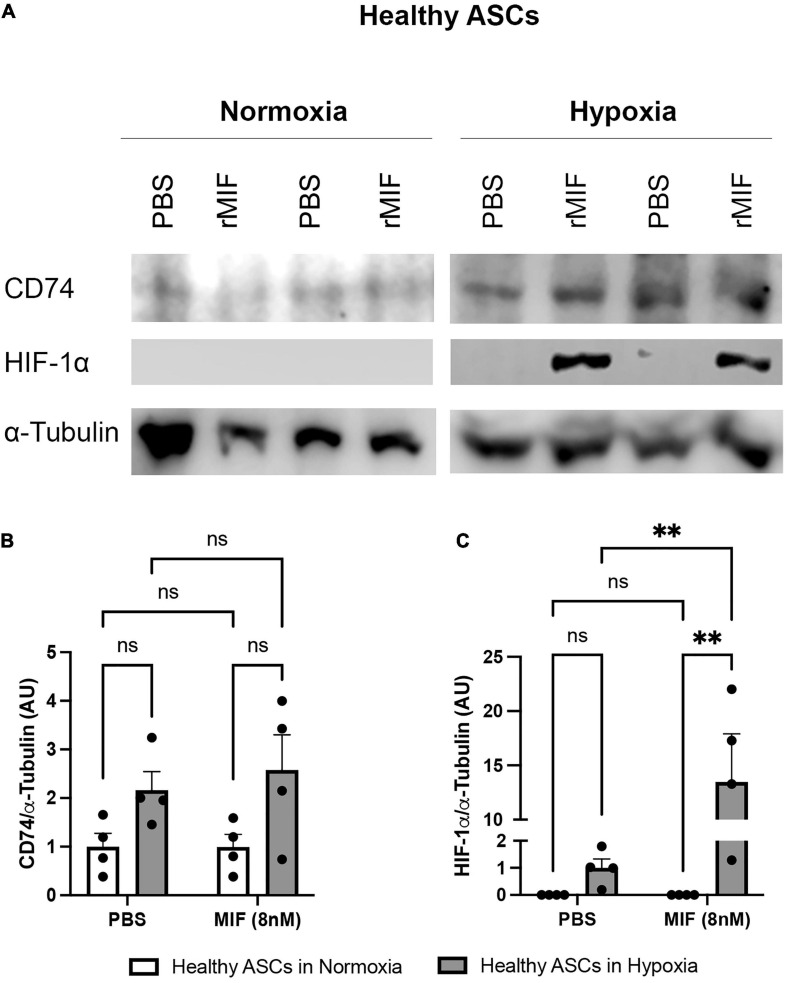
HIF-1α and CD74 expression after MIF stimulation in normoxic and hypoxic ASCs. ASCs from healthy donor sites (Group A) were stimulated with 100 ng/ml of recombinant MIF and cultured in growth medium at normoxic oxygen levels of 21% O_2_ or hypoxic oxygen levels of <1% O_2_. Control cells (0 ng/ml) received PBS as vehicle. Cells were lysed 24 h after induction of hypoxia and Western blot analysis was performed and evaluated using Image J software. Tubulin was detected for total protein standardization. **(A)** Representative blots of HIF-1α and CD74 expression in normoxic and hypoxic ASCs are depicted. Analyses were performed on four biological replicates (*n* = 4). Band intensities were quantified and graphically displayed as **(B)** relative CD74/tubulin and **(C)** relative HIF-1α /tubulin and normalized to the respective control group (PBS + normoxia). Data are mean values ± SEM (two-way ANOVA, Holm-Šídák’s multiple comparisons test, ^∗∗^*p* < 0.01).

It was shown earlier that HIF-1α induces MIF expression (Welford et al., 2006; Simons et al., 2011), while MIF amplifies HIF-1α stabilization, prompting a positive feedback that leads to further MIF expression (Winner et al., 2007). In a hypoxic environment, MIF mediates HIF-1α expression by stimulation of its cognate receptor CD74 and activation of the ERK/mammalian target of rapamycin (mTOR) pathway (Gaber et al., 2011). To study a possible link between secreted, exogenous MIF, CD74, and HIF-1α expression and the proliferation of ASCs, we assessed HIF-1α and CD74 levels in normoxic and hypoxic ASCs treated with recombinant MIF (Figure 8). There was a trend toward increased levels of CD74 under hypoxic conditions, while CD74 expression was unaffected by recombinant MIF (Figure 8B). HIF-1α is not stabilized in normoxic ASCs whereas low volumes were detectable under hypoxia. Of note, recombinant MIF also led to a substantial significant up-regulation of HIF-1α (>10-fold) compared to vehicle-treated control cells (*p* < 0.01) (Figure 8C). The data suggest that exogenous MIF stabilizes HIF-1α, an effect that might mechanistically be facilitated by CD74 under hypoxic conditions.

### Recombinant MIF Reduces IL-1β Secretion From ASCs

Soluble factors released from ASCs have been recognized as a major mode of action of ASCs (Salgado et al., 2010). The secretome of ASCs can be profoundly boosted by conditioning ASCs with hypoxic or inflammatory stimulus.

**FIGURE 9:**
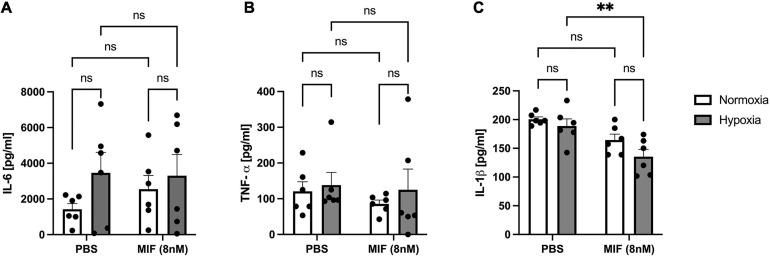
Release of IL-6, TNF-α, and IL-1β from ASCs from healthy donor site (Group A) following hypoxia and stimulation with recombinant human MIF. Cells were stimulated with 100 ng/ml of recombinant MIF and cultured in growth medium at normoxic oxygen levels of 21% O_2_ or hypoxic oxygen levels of <1% O_2_. Supernatants of ASCs were collected at 24 h after induction of hypoxia and ELISA was performed to assess cytokine secretion by ASCs under normoxic and hypoxic conditions. Results are displayed for **(A)** IL-6 (*n* = 6), **(B)** TNF-α (*n* = 6), and **(C)** IL-1β (*n* = 6). Data are mean values ± SEM (two-way ANOVA, Holm-Šídák’s multiple comparisons test, ^∗∗^*p* < 0.01).

Because MIF has an established role as an upstream regulator of multiple cytokines (Calandra and Roger, 2003) and is rapidly released following hypoxia, we hypothesized that MIF may influence the release of other soluble factors and thereby modulate the paracrine capacity of ASCs. Following stimulation with recombinant MIF under normoxic and hypoxic conditions, we measured the secretion of IL-6, TNF-α, and IL-1β, all of which have been shown to be secreted by MSCs (Paquet et al., 2015; Antebi et al., 2018) and to be downstream targets of MIF (Calandra and Roger, 2003). Under the conditions used in our experimental set-up, neither recombinant MIF nor hypoxia showed an effect on IL-6 or TNF secretion of ASCs, although there was a trend toward higher released IL-6 levels in incubations with hypoxic versus normoxic ASCs, (Figures 9A,B). Interestingly, the secretion of IL-1β was significantly decreased by recombinant MIF treatment, both under normoxic and hypoxic conditions, while hypoxia itself had no significant effect (Figure 9C).

### Paracrine/Autocrine MIF Activity Is Involved in TNF-α Release From ASCs Under Hypoxic Conditions

To further study the role of MIF in the release of the cytokines IL-6, TNF-α, and IL-1β in ASCs following hypoxia and to better understand its paracrine or autocrine activity, ASCs obtained from chronic non-healing wounds were treated with a blocking MIF antibody and cultured under hypoxic conditions. Supernatants of ASCs were collected 24 h after induction of hypoxia, and ELISAs performed to assess cytokine secretion.

**FIGURE 10:**
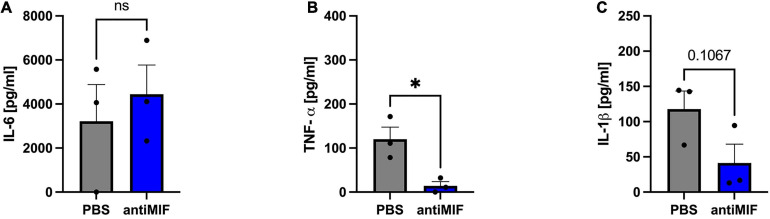
Release of IL-6, TNF-α, and IL-1β from ASCs from chronic non-healing wounds (Group B) following hypoxia and neutralization of exogenous MIF via an anti-MIF antibody. Cells were treated with anti-MIF antibody (NIH/IIID.9) and cultured in growth medium at hypoxic oxygen levels of <1% O_2_. Supernatants of ASCs were collected at 24 h after induction of hypoxia and ELISA were performed to assess cytokine secretion by ASCs under normoxic and hypoxic conditions. Results are displayed for **(A)** IL-6 (*n* = 3), **(B)** TNF-α (*n* = 3), and **(C)** IL-1β (*n* = 3). Data are mean values ± SEM (unpaired, two-sided *t*-test, not significant). ^∗^*p* < 0.05.

While IL-6 levels remained unaffected following neutralization of paracrine MIF in hypoxic ASCs from chronic non-healing adipose tissue (Figure 10A), TNF-α levels were strongly reduced (*p* < 0.05) (Figure 10B). IL-1β levels were also reduced, but this effect did not reach statistical significance (*p* < 0.11) (Figure 10C). Comparable levels of IL-6 and TNF-α released from ASCs from healthy and inflamed donor sites following hypoxia (Figures 9A,B versus Figures 10A,B) confirm that IL-6 and TNF-α secretion remains unaffected by higher concentrations of exogenous MIF. Furthermore, secreted levels of IL-1β are comparable between hypoxic ASCs isolated from adipose tissue of chronic non-healing wounds and hypoxic ASCs from healthy donor sites stimulated with recombinant MIF, which constitutes the experimental equivalent to wound ASCs, while the control group (hypoxia + PBS) seem to secrete higher amounts of IL-1β. In aggregate, these findings may suggest that IL-1β secretion is down-regulated in ASCs from chronic non-healing wounds (Figure 9C versus Figure 10C).

## Discussion

Tissue hypoxia is a condition of insufficient oxygen supply that is found in several conditions including critical wounds. Adipose tissue from subcutaneous layers adjacent to chronic non-healings wounds may experience prolonged hypoxia due to reduction in blood perfusion and other factors (Sen, 2009). Key cells in the context of adipose tissue and wound hypoxia are ASCs, stem cells of mesenchymal origin with considerable reparative functions. The pleiotropic adipokine and cytokine MIF has been identified in different entities of adipose tissue inflammation including obesity and wound healing, which share a state of tissue hypoxia (Semenza, 2003; Brahimi-Horn and Pouyssegur, 2007). Moreover, MIF was shown to be up-regulated under hypoxia in endothelial and cancer cell lines (Oda et al., 2008; Simons et al., 2011). This study served to elucidate the effects of hypoxia and MIF on human ASCs derived from healthy donor sites and chronic non-healing wounds. We found that hypoxia did not induce proliferation/viability of ASCs but led to an increased MIF expression and secretion as well as CD74 expression over time. Interestingly, a distinct increase in MIF secretion was observed in ASCs harvested from chronic non-healing wounds when compared to ASCs from healthy donor sites. Elevated MIF secretion under hypoxia was paralleled by altered ERK signaling, at an early hypoxia induction time point of 4 h. Yet, recombinant MIF did not have any effect on ASC viability under hypoxia, although it contributed to the up-regulation of stabilization and/or expression of HIF-1α. The addition of exogenous MIF significantly reduced IL-1β secretion from ASCs, while IL-6 and TNF-α remained unaffected.

In regenerative medicine, ASCs attract great interest as a cell source for clinical applications owing to their high reparative capacity. More than that, the *in vivo* stem cell niche is characterized by oxygen levels below 2% in humans (Mohyeldin et al., 2010), whereas non-physiologically high oxygen levels generate exogenous oxidative stress and cell senescence (Estrada et al., 2012). Consequently, the question arises how tissue hypoxia influences properties of ASCs and if exogenous factors such as MIF may have a beneficial effect. We first sought to investigate the effect of hypoxic culture conditions on ASC viability when compared to normoxia. While previous studies reported controversial effects of hypoxia on the viability of various cell types including MSCs (Wang et al., 2005; Estrada et al., 2012; Yamamoto et al., 2013; Choi et al., 2014), the present study demonstrated that hypoxic treatment at <1% O_2_ for 72 h does not enhance ASC proliferation. The proliferation behavior of ASCs isolated from healthy adipose tissue and chronic non-healing wounds was similar under normoxia and hypoxia. As a limitation, our measurements were restricted to 72 h and therefore evaluation of a potential effect of hypoxic conditions beyond that time frame was not possible. It was shown that ASCs under hypoxic culture for one or 2 weeks exert pro-proliferative effects of hypoxia (Wang et al., 2005; Estrada et al., 2012; Yamamoto et al., 2013; Choi et al., 2014). Decreased proliferation of human MSCs in hypoxic culture was linked to a resting state, indicating decelerated cell division and differentiation (Holzwarth et al., 2010).

We found significantly elevated MIF secretion and expression after hypoxic challenge in ASCs isolated from non-healing chronic wounds, whereas ASCs from healthy donor sites did not increase MIF secretion upon hypoxia. These results support our previous finding that MIF expression is associated with hypoxia, and that MIF is over-expressed in non-healing wounds (Calandra and Roger, 2003; Wang et al., 2007; Kim et al., 2015b). Furthermore, our observations indicate that hypoxia triggers MIF-expression/secretion more easily in cells that already were exposed to an inflammatory status in the past. The underlying mechanisms are subject to further investigations.

Next, the expression of CD74 as one of the receptors involved in MIF-driven pathways of inflammation as well as proliferation and protection was examined (Leng et al., 2003). An elevation of MIF secretion by ASCs from chronic non-healing wounds upon hypoxia was accompanied by an up-regulated MIF and CD74 expression. To gain an insight into MIF-CD74-dependent signaling, we analyzed PI3K/AKT and MAPK/ERK pathways that are involved in cell survival and apoptosis in response to hypoxia (Leng et al., 2003; Lue et al., 2006, 2007). Skin injury models have suggested that the cutaneous healing capacity of hypoxia-conditioned MSCs may be mediated via PI3K/AKT pathways (Jun et al., 2014). MIF-dependent AKT signaling protects against reperfusion injury in the ischemic heart (Zernecke et al., 2008; Pohl et al., 2016), whilst it delays cellular senescence and enhances multipotency of MSCs (Palumbo et al., 2014; Xia et al., 2015). Interestingly, ERK phosphorylation was increased under hypoxia, while no significant changes of AKT signaling were observed. Missing effects on AKT activation in our experiments may be explained by our limited measurement time points that do not cover the early AKT activation phase and the limited number of studied specimens. Altered ERK signaling under hypoxia is consistent with previously reported sustained ERK signaling (Mitchell et al., 1999). We hypothesize that the hypoxia-induced MIF secretion may be linked with an altered CD74/ERK signaling response in ASCs, which in turn may activate HIF-1α.

Migration inhibitory factors role in cell migration, cell proliferation, wound repair and many more fundamental cell functions is well documented (Abe et al., 2000; Zhao et al., 2005; Dewor et al., 2007). To investigate whether an increase of MIF upon tissue hypoxia may be a paracrine/autocrine protective mechanism to stimulate ASC viability and to evaluate MIF’s role as a potential agent to treat ASCs (e.g., in oxygen-depleted wounds), recombinant MIF was added to ASCs under hypoxia. First, we assessed the activation of ERK and AKT, which both exert pro-survival and pro-proliferative effects on multiple cell types. Following stimulation with increasing concentrations of recombinant MIF, a tendency toward a dose-dependent phosphorylation of AKT and ERK was observed. Yet, stimulation with increasing concentrations of recombinant MIF did not increase the viability of hypoxic ASCs when compared to the unstimulated hypoxic control. However, this observation is in line with our other findings that hypoxic ASCs from chronic-non-healing wounds, which secreted significant amounts of MIF, exhibited no increased proliferation when compared to hypoxic ASCs from healthy adipose tissue, which only released basal levels of MIF. It was shown earlier that HIF-1α induces MIF expression (Welford et al., 2006; Simons et al., 2011), while MIF amplifies HIF-1α stabilization via the CD74/MAPK/PI3K pathway (Gaber et al., 2011), prompting a positive feedback that leads to further MIF expression (Winner et al., 2007). An enhanced proliferation of hypoxic ASCs has been demonstrated to involve HIF-1α activation, FGF2 production and activation of ERK1/2 and AKT (Kakudo et al., 2015). To elucidate a possible link between secreted exogenous MIF, CD74, HIF-1α expression and ASC proliferation, we assessed HIF-1α and CD74 levels in normoxic and hypoxic ASCs treated with recombinant MIF. In accordance to previous findings, we demonstrated that HIF-1α is stabilized in ASCs subjected to hypoxia (Stubbs et al., 2012; Yamamoto et al., 2013; Kakudo et al., 2015), and that HIF-1α levels were synergistically amplified in the presence of recombinant MIF (Fu et al., 2010; Gaber et al., 2011). In contrast, CD74 levels remained stable following treatment with hypoxia and recombinant MIF. Because we observed an enhanced HIF-1α stabilization as well as a possible AKT and ERK activation following stimulation with recombinant MIF, our data might confirm previous results that MIF/CD74 interaction enhances HIF-1α stabilization through activation of ERK and AKT pathway (Gaber et al., 2011). Previously, HIF-1α activation, FGF2 production and activation of ERK1/2 and AKT has been implicated to mediate enhanced proliferation of human ASCs under hypoxia (Kakudo et al., 2015). Although, we showed that recombinant MIF enhances stability of HIF-1α and might dose-dependently phosphorylate ERK1/2 and AKT, we did not observe an enhanced proliferation of ASCs subjected to recombinant MIF and hypoxic tension. In summary, based on our experiments MIF-induced HIF-1α stabilization appears not to culminate in an increased proliferation rate of ASCs under hypoxia.

Recently, the paracrine action of ASCs has been recognized as the key mechanism by which ASCs exert their regenerative potential (Salgado et al., 2010; Frese et al., 2016). In experimental tissue engineering studies, for instance, it was shown that implanted ASCs quickly disappeared within a few days but attracted immune cells which promoted subsequent reparative effects (Roh et al., 2010). Since MIF acts as an upstream regulator of multiple cytokines (Calandra and Roger, 2003), we investigated the secretion profile of IL-6, IL-1β, and TNF-α, which have been shown to be secreted by MSCs (Paquet et al., 2015; Antebi et al., 2018) and to be downstream of MIF (Calandra and Roger, 2003). The profile of soluble factors released by MSCs is profoundly influenced by sex, age, species, underlying disease conditions and environmental factors like hypoxia or other cytokine stimuli (Malhotra et al., 2021).

Interleukin-6 is a pleiotropic cytokine with various inflammatory but also homeostatic effects, IL-6 released from ASCs has been demonstrated to promote angiogenesis and wound healing in a paracrine fashion (Heo et al., 2011; Pu et al., 2017). Previous studies have shown that IL-6 secretion can be triggered by several stimuli, including hypoxia (Tamm et al., 1998) and MIF-induced CD74 activation (Xiong et al., 2014). In our study, we detected an almost two-fold increase of soluble IL-6 released from hypoxic ASCs when compared to normoxic control. However, the difference was not significant due to high sample to sample variation, which may be explained by the heterogenous characteristics of donors, including age, sex and different donor sites of adipose tissue. The stimulation with recombinant MIF did not induce an increased IL-6 release when compared to the control group. Additionally, blocking exogenous MIF in hypoxic ASCs from chronic non-healing adipose tissue did not diminished IL-6 levels in the supernatant. Furthermore, comparable levels of IL-6 between ASCs from healthy and inflamed donor sites following hypoxia confirm the results of the MIF stimulation experiments that IL-6 secretion remains unaffected by higher concentrations of exogenous MIF. This indicates that IL-6 release from ASCs is mostly regulated by hypoxia rather than by MIF.

Previous studies reported that MSCs secrete TNF-α and IL-1β (Harrell et al., 2019) and that their paracrine action is involved in immunomodulatory effects (Malhotra et al., 2021). Furthermore, TNF-α and IL-1β are commonly used to precondition of MSCs and ASCs as they profoundly modulate their secretome (Lee et al., 2010). In our work, TNF-α was continuously secreted independent of applied stimuli (hypoxia or recombinant MIF alone or in combination), while IL-1β was significantly decreased following treatment with recombinant MIF. However, blocking the paracrine/autocrine activity of endogenous MIF in hypoxic ASCs from chronic non-healing adipose tissue significantly reduced TNF-α levels in the supernatant. Although not significant, IL-1β also showed a tendency toward reduced secretion following neutralization of exogenous MIF. Furthermore, similar levels of TNF-α as well as lower levels of IL-1β released from hypoxic ASCs from inflamed donor sites compared to hypoxic ASCs from healthy donor sites confirm the results of the MIF stimulation experiments that IL-1β secretion is reduced by pathophysiological external MIF levels, while TNF-α secretion remains unaffected by pathophysiological external MIF. Accordingly, Meyer-Siegler et al. (2004) reported that anti-MIF antibody treatment significantly reduced mRNA expression and secretion of TNF-α in human bladder cancer cells. Thus, our results demonstrate that exogenous MIF is required for the expression and/or secretion of TNF-α in ASCs, while supplementing supra-physiological concentrations of recombinant MIF did not enhance TNF-α release. One may hypothesize that exogenous MIF, regardless of its concentration, maintains low basal amounts of exogenous TNF-α, which in turn triggers the release of soluble factors from ASCs. Regarding the secretion of IL-1β, a recent study from Lang et al. (2018) has shown that intracellular MIF is involved in the assembly and activation of the NLRP3 inflammasome which subsequently triggers IL-1β release. However, stimulation of *Mif*^–/–^ BMDMs treated with recombinant MIF did not facilitate the NLRP3 inflammasome activation, which indicated a role for cell-intrinsic, intracellular MIF. Furthermore, the transcription of IL-1β is neither affected by inhibition or depletion of intracellular MIF nor by neutralization of exogenous MIF (Meyer-Siegler et al., 2004), In contrast to findings of Lang et al. (2018) who reported that recombinant MIF cannot restore IL-1β levels in *Mif*-deficient cells, our current findings and the results from Meyer-Siegler et al. (2004) indicate that blocking exogenous MIF significantly inhibits IL-1β secretion. A possible but to our knowledge not yet proven mechanism may be a MIF-dependent activation of protein kinase A (PKA) (Mitchell et al., 1999; Shi et al., 2006), which in response inhibits the inflammasome assembly by phosphorylation of human NLRP3 at Ser295 (Mortimer et al., 2016; Caseley et al., 2020). Whether reduced IL-1β levels may be beneficial for the maintenance of biological activity of ASCs itself or for the modulation of cellular functions of other cell types via a paracrine action remains to be elucidated.

Our study has limitations. First, we did not study effects on other cellular functions such as differentiation or migration, and other cell types such as resident cells in the subcutaneous adipose tissue layer (e.g., macrophages, adipocytes). Second, a major limitation of this study is the low number of patient samples. Particularly the harvest of sufficient volumes of adipose tissue from patients with chronic non-healing wounds and the subsequent harvest are challenging. While considerable amounts of adipose tissue were needed for the experiments, the debridement of chronic wounds has to be carefully performed and mostly includes only infected or necrotic adipose tissue of which a great part again serves as samples for clinical diagnostics. Thus, the majority of the collected tissue had to be discarded due to insufficient cell numbers eventually. Additionally, despite careful sample preparation, cell culture contamination was observed frequently.

## Conclusion

Taken together, MIF secretion and CD74 expression levels were significantly up-regulated in ASCs from chronic non-healing wounds under hypoxia. Although, exogenous MIF enhanced HIF-1α stabilization in ASCs subjected to hypoxia, which possibly involves the interaction of CD74 and downstream activation of ERK and AKT, the viability of hypoxic ASCs remained unaffected by exogenous MIF in general. A recently growing body of evidence suggests that the ASC’s primary way of action is exerted by the release of soluble factors rather than differentiation and proliferation. In line with this assumption, we propose that increased levels of MIF, such as found in ASCs from chronic non-healing wounds, may promote tissue repair by modulating the release of soluble factors and the reduction of IL-1β by recombinant MIF supplementation. However, the precise regulatory effects of MIF on the secretome of ASCs is elusive and requires further investigation.

## Data Availability Statement

The raw data supporting the conclusions of this article will be made available by the authors, without undue reservation.

## Ethics Statement

The studies involving human participants were reviewed and approved by RWTH Aachen University (EK 213/17), Cantonal Ethics Committee Zurich (BASEC-Nr 2019-00389). The patients/participants provided their written informed consent to participate in this study.

## Author Contributions

NP, JB, and B-SK: conceptualization, software, validation, and resources. EH, JS, SG, GS, YT, MB, and B-SK: methodology. EH, JS, TR, EG, YT, MB, RS, NL, and B-SK: formal analysis. EH, JS, and B-SK: investigation. EH, JS, and SG: data curation. EH, JS, S-YS, and B-SK: writing–original draft preparation. EH, JS, TR, EG, RS, S-YS, NL, NP, JB, and B-SK: writing–review and editing. EH and B-SK: visualization. JB, NP, and B-SK: supervision. B-SK: project administration. JB and B-SK: funding acquisition. All authors have read and agreed to the published version of the manuscript.

## Conflict of Interest

The authors declare that the research was conducted in the absence of any commercial or financial relationships that could be construed as a potential conflict of interest.

## Supplementary Material

Supplementary Figure 1 Adipose-derived stem cell (ASC) characterization for osteogenic, chondrogenic, and adipogenic differentiation. The trilinear differentiation of isolated ASCs was measured *in vitro* be exposing ASCs to osteogenic, chondrogenic and adipogenic differentiation media as reported earlier (Yoshinoya et al., 2020). Osteogenic differentiation was determined by Alizarin red staining (top), chondrogenic differentiation by Alcian blue staining (middle) and adipogenic differentiation by oil red O staining (bottom).

Supplementary Figure 2 Adipose-derived stem cell (ASC) characterization by flow cytometry. The surface marker expression of ASCs was examined by flow cytometry. ASCs were defined as cells with no expression of the endothelial marker CD31 and hematopoietic marker CD45. ASCs were positive for the stem cell markers CD34, CD73, CD90, and CD105 to various degrees.
