# Supplementary figures and images for: The Role of Macrophage Migration Inhibitory Factor in Adipose-Derived Stem Cells Under Hypoxia
Source: Front Physiol. 2021 Jul 21;12:638448. doi: 10.3389/fphys.2021.638448 (PMC8334873; doi:10.3389/fphys.2021.638448)

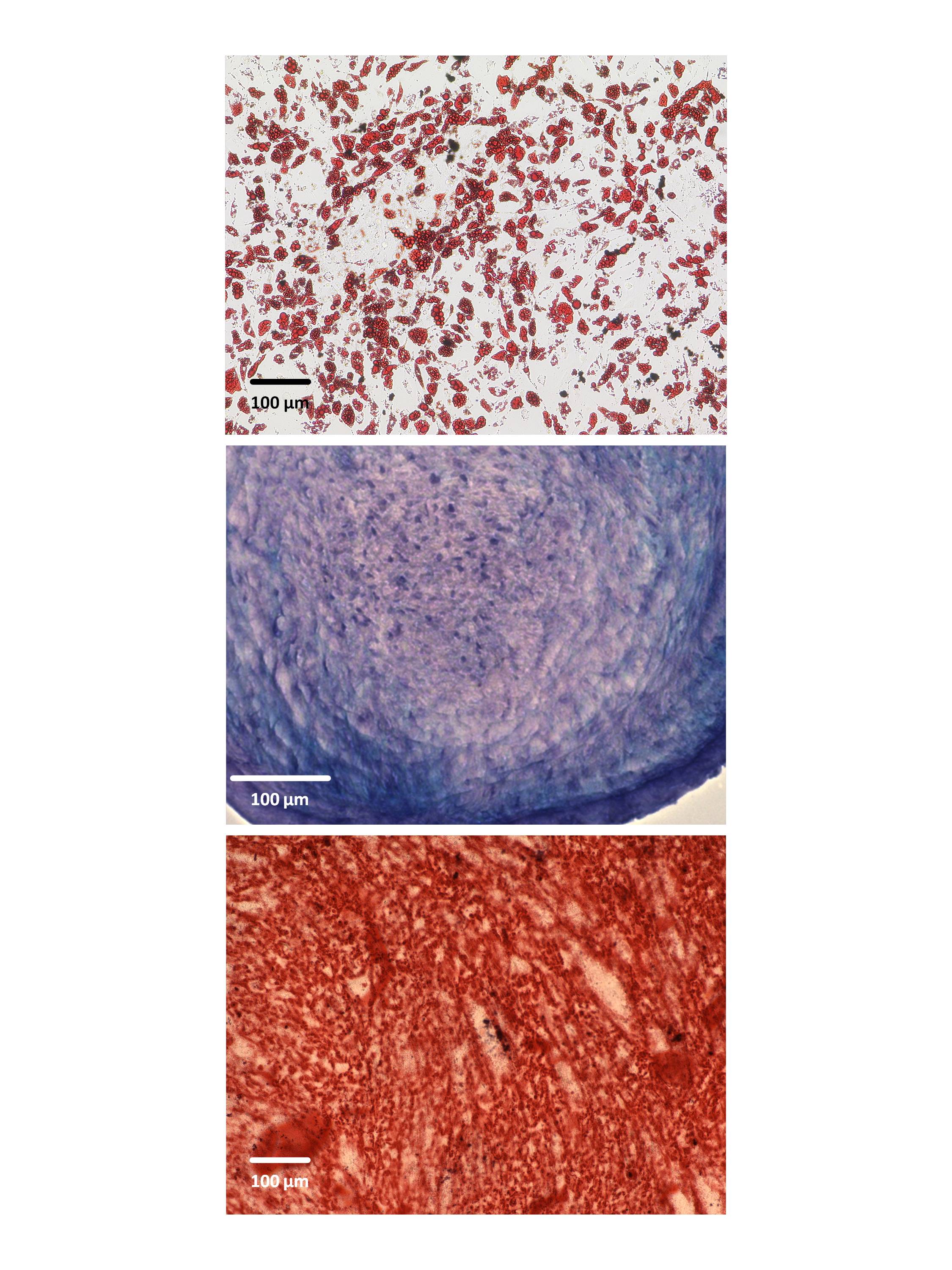

Supplement: Supplementary Figure 1 — Adipose-derived stem cell (ASC) characterization for osteogenic, chondrogenic, and adipogenic differentiation. The trilinear differentiation of isolated ASCs was measured in vitro be exposing ASCs to osteogenic, chondrogenic and adipogenic differentiation media as reported earlier (Yoshinoya et al., 2020). Osteogenic differentiation was determined by Alizarin red staining (top), chondrogenic differentiation by Alcian blue staining (middle) and adipogenic differentiation by oil red O staining (bottom). [file Image_1.jpg]

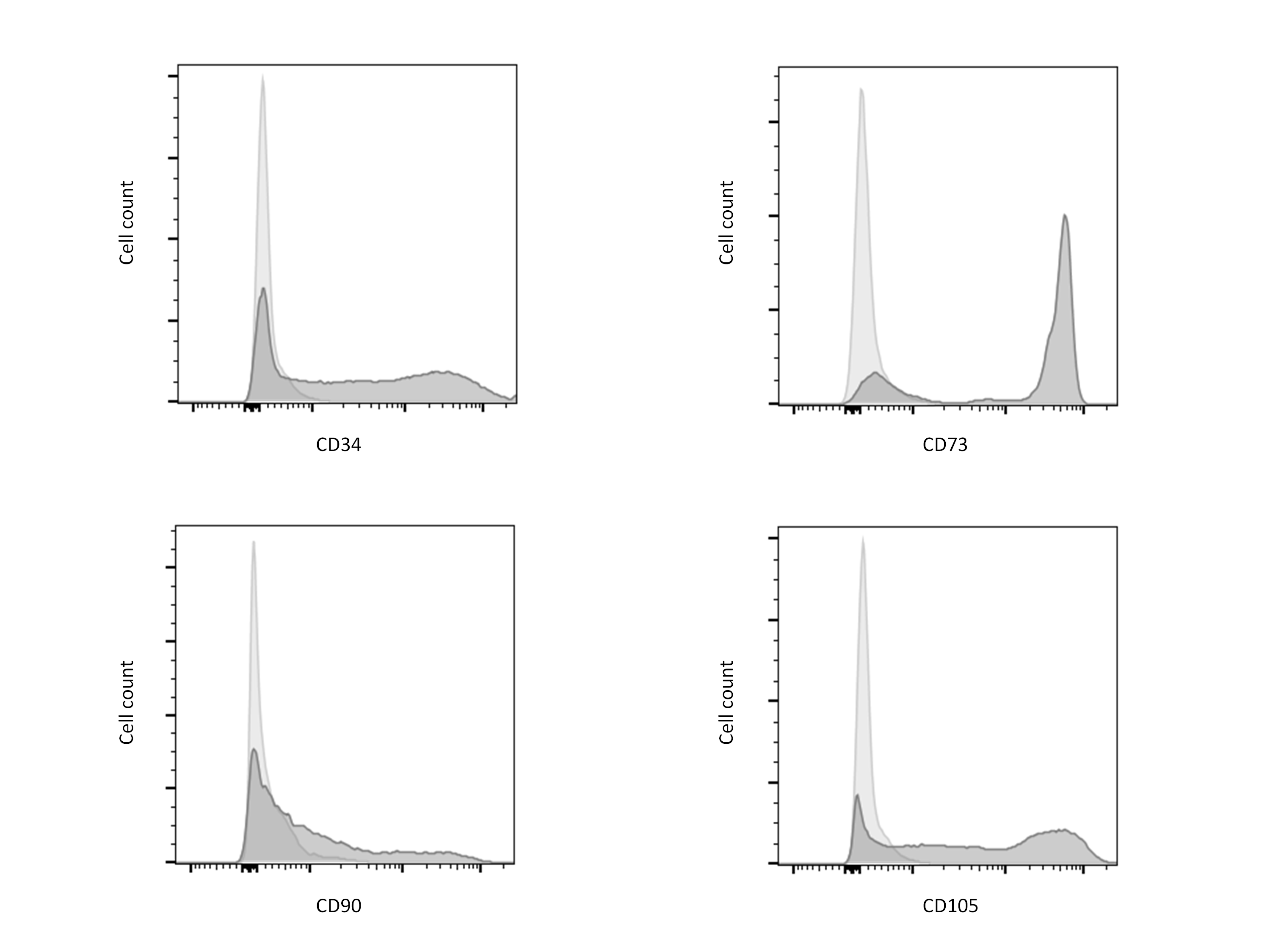

Supplement: Supplementary Figure 2 — Adipose-derived stem cell (ASC) characterization by flow cytometry. The surface marker expression of ASCs was examined by flow cytometry. ASCs were defined as cells with no expression of the endothelial marker CD31 and hematopoietic marker CD45. ASCs were positive for the stem cell markers CD34, CD73, CD90, and CD105 to various degrees. [file Image_2.tif]
